# Thermodynamic quantities and Urmia Sea water evaporation

**DOI:** 10.1186/1746-1448-6-3

**Published:** 2010-03-31

**Authors:** Nosrat Heidari, Mina Roudgar, Neda Ebrahimpour

**Affiliations:** 1 Department of Chemistry, Faculty of Sciences, Urmia University, Urmia 57159-165, Iran

## Abstract

The relation between climatic parameters (relative air humidity) and the water activity of the Urmia Sea water determines the possible maximum evaporation of the lake. Using the Pitzer thermodynamic approach, the activity of the Urmia Lake water during evaporation was calculated and compared to the present relative air humidity above the water. Present climatic conditions allow the Urmia Sea water to evaporate down to water with activity of 0.55, corresponding to the lowest air humidity measured over the lake. This water activity falls in the range of halite precipitation, while carnalite precipitation starts at somewhat lower (a H_2_O = 0.499) point. Our dynamic model predicts that for air humidity as low as 55% (reflecting present climate conditions), the Urmia Sea level may drop to as low as 1270 m (i. e., 1270 m above mean sea level). At that point, the lake water volume will have a volume of 11 km^3^. For the sake of comparison, at the beginning of 1990, the level of the lake was 1275 m, its volume was 25 km^3^, and its surface area was 5145 km^2^.

## Introduction

Lake Urmia (or Orumiyeh), is one of the largest permanent hypersaline lakes in the world and resembles the Great Salt Lake in the western USA in many respects of morphology, chemistry and sediments [1]. Despite this, and its several values, including conservation, little literature has been published on the lake and its biota [2-7]. The predominance of the Na^+ ^and Cl^- ^ions illustrates the thalassohaline character of Urmia lake [8]. Therefore, Urmia Lake is an oligotrophic lake of thalassohaline origin [9] with an ionic strength between 5.5-7.5, located in northwestern Iran at an altitude of 1275 m above sea level. The total surface area ranges between 4750 km^2 ^and 6100 km^2 ^[10] depending on evaporation and water influx. The maximum length and width of the lake are 128-140 km and 50 km, respectively [10,11]. The average and maximum depths are 6 m and 16 m, respectively [12]. The Urmia lake is not a homogeneous body of water. There are horizontal variations in temperature and salinity but these are too small to make a difference on a climatic scale [13].

The air temperature usually ranges between 0 and -20°C in winter, and up to 40°C in summer [14]. From this point of view, Urmia Lake is a critical asset for the region, because it acts to moderate these extremes [15].

Hydrologic conditions are extremely important for the maintenance of a given water body's structure and function and affect many abiotic factors which, in turn, may impact the biota that develop in it [16].

Because saline lakes occur primarily in endorheic basins, they may be particularly sensitive to environmental changes because their size, salinity and annual mixing regimes vary with alterations in their hydrologic budgets [17,18].

The main cations in the lake water include Na^+^, K^+^, Ca^+2^, and Mg^+2^, while Cl^-^, SO_4_^-2^, HCO_3_^- ^are the main anions [19]. Sodium ions are at slightly higher concentration in the south compared to the north of the lake, which could result from the shallower depth in the south, and a higher net evaporation rate [20,21].

The current study attempts to obtain an answer by modeling the physico-chemical coupling between evaporation and mineral precipitation. A thermodynamic model based on the Pitzer ion interaction approach [22-24] has been level oped (see Appendix A) for a quantitative estimate of water activity variation and mineral precipitation in the Urmia Lake. The developed model was used to determine the final stage of the evaporation of the Urmia lake as a function of the present day climate of relative air humidity over the lake.

Kinsman [25] was the first to propose that it is the mean relative humidity in the atmosphere above evaporitic basins that controls their final salinity and ultimate mineral fades. In his paper, Kinsman used available experimental data as well as his field observations from the Persian Gulf. It is clear that evaporation from the Urmia Lake can not continue indefinitely. Due to evaporation, the ionic strength of the water increases while water activity in the liquid phase decreases.

When chemical potentials of water in the lake's surface layer and the adjacent air layer become equal, evaporation ceases. The present study provides a predictive model, which spans a much larger range of water activity values then those based on the experimental data used by Walton [26], but it is our understanding of those objections that in Wallton's view, Kinsman's simplifications prevent his theory from being all encompassing and, by making some simplifications of his own, Walton produces some very particular situations (e.g., large brine pools, but not too large, close to the sea shore) where Kinsman's theory is bound to fail.

It is not our intention to trace the detailed variations in the meteorological parameters (radiation, temperature, pressure, etc.) but to calculate the final stages of evaporation which the Urmia lake can reach at different humidities, at a temperature of 25°C. The proposed method of calculation is strictly thermodynamic, and is not intended to estimate the time needed to achieve equilibrium between the brine and the overlying atmosphere.

### The process of the Urmia lake water evaporation

Evaporations of the Urmia lake water will continue as long as the chemical potential of water in the surface layer () is larger than that of the overlying air layer (). When these quantities become equal, evaporation stops:(1)

Hence,(2)

If the air and the water surface temperatures are equal (which is the case at air-sea interface), Eq. (2) simplifies to:(3)

Krumgalz and Millero [28-30] demonstrated that the water activity of hypersaline sea such as Dead Sea water () could be calculated precisely by the Pitzer ion interaction approach [23]. The water activity of air is defined as:(4)

Where *P*^*air *^is the water vapor pressure in air at a given temperature, and  is the pressure of the saturated vapor at the same temperature. According to this definition, water activity in air is the relative air humidity.

The initial chemical composition of the Urmia Lake used for the thermodynamic modeling is represented in Table 1. Using the above-mentioned approach, we computed the water activity of the Urmia Lake water and its derivatives at various temperatures (Table 2).

**Table 1:** Chemical composition of Urmia lake water derivatives obtained by modelling the isothermal evaporation of Urmia lake water at 25°C and at the starting point of mineral precipitation

Ions	**Urmia Sea water (mol/kg H**_2_**O)**	**Initial point of halite (NaCl) Precipitation (mol/kg H**_2_**O)**	**Initial point of carnalite (KMgCl**_3_**.6H**_2_**O) Precipitation (mol/kg H**_2_**O)**
Na^+^	4.900	5.6457	3.78
K^+^	0.005	0.005759	0.018
Mg^2+^	0.46125	0.5314	1.62
Ca^2+^	0.015625	0.018	0.055
Cl^-^	5.400	6.22105	15.3
Br^-^	0.00625	0.0072	0.022
*HCO*_3_^-^	0.0098	0.011295	0.343
*SO*_4_^-^	0.18125	0.0834	0.0494
l	6.477	7.211	13.03
*f*_*enrich*_	1	1.1525	3.5
*α*H_2_O	0.775	0.741	0.499
*ρ*(g/cm^3^)^a^	1.2030	1.2142	1.3186

^a ^The density values were calculated by the method described in Krumgals et al. [27]

Table 2 demonstrates that there is no significant temperature influence on the water activity of various Urmia Lake water derivatives. Therefore we used 25°C for the ensuing computations The dynamic modeling of the isothermal evaporation of the Urmia Sea was based on the following:

**Table 2:** Water activity of Urmia sea water and its derivations at various temperatures

Brine	Temperature (°C)					
	
	10	20	25	30	40	50
Urmia Sea water	0.698	0.771	0.775	0.779	0.781	0.784
Evaporated Urmia Sea water in the halite point	0.736	0.739	0.741	0.743	0.750	0.753
Evaporated Urmia Sea water in the carnalities point	0.490	0.494	0.499	0.507	0.510	0.522

^a ^The density values were calculated by the method described in Krumgals et al. [27]

1-The local climate in the region of the Urmia Sea is steady state and is not expected to change drastically; and

2-the Urmia Sea surface layer is chemically homogeneous throughout the lake.

The evaporation degree can be characterized by an enrichment factor (*f*_*enrich*_) defined as:(5)

Where  and  represent the mass of water in the basin at any specific time, and the mass of water removed from the basin during a given period of evaporation, Removal of water from the lake is mainly carried out by evaporation and, to a lesser extent, by precipitation of hydrated minerals such as carnallite (KMgCI_3_. 6H_2_O).

**Figure 1:**
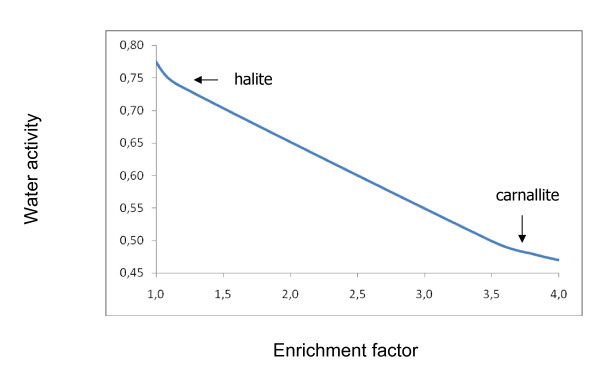
**Water activity changes for isothermal evaporation of Urmia Sea water at 25°C**. The initial precipitation points of halite and carnallite, computed in accordance to the Pitzer approach are indicated.

The results of calculated dynamic isothermal evaporation of the Urmia Sea at 25°C are illustrated in Fig. 1, which shows the variation  vs. *f*_*enrich*_. The two arrows indicate the onset points of halite responding to these events (enrichment factor, water activity, ionic concentration and density of brines corresponding to starting points of halite and carnallite precipitation) are listed in Table 1. Thus, the water activity of the initial Urmia lake water is 0.775 and decreases upon evaporation to 0.741 at the halite point and to 0.499 at the carnallite point.

### water activity relations between Urmia sea water and atmosphere

Water activity in the Urmia Lake is most of the time larger than that in the air. Therefore, evaporation from the lake must have been the rule. Although possible [31], it is unlikely that condensation of water vapor of the lake occurred, except on rare occasions. The volume of the Urmia Lake (*v*_*US*_) at any evaporation stage can be determined as:(6)

Where *V*_*US brine *_is the volume of residual Urmia Sea brine and  is the sum of the mineral volumes precipitated at a particular enrichment factor.

The residual volume of the Urmia Lake brine is given by:(7)

Where *G*_*USW *_is the mass of water in the Urmia Lake at the initial period,  is the mass of water evaporated at a particular stage of evaporation, ↓ *g*_min *erals *_is the mass of salts precipitated during the same period and *ρ*_brine _is the density of the residual brine.

**Figure 2:**
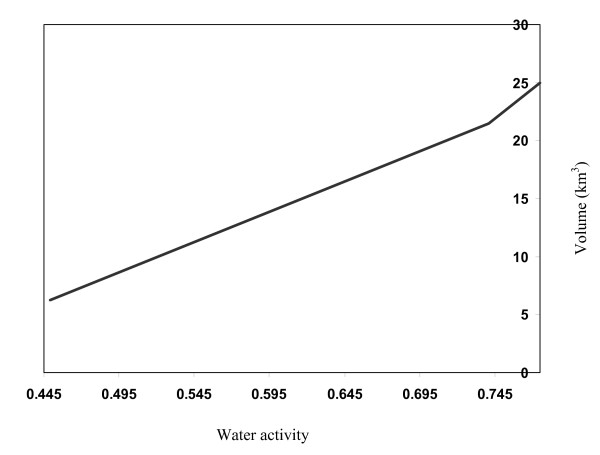
**Volume of Urmia sea vs**. Water activity during isothermal evaporation.

The above model was applied to the water activity range of 0.3-0.7. It follows that Urmia Lake water can evaporate down to a water activity of 0.55, corresponding with the lowest air humidity over the lake under present climate conditions. During evaporation, the water activity remains within the range of halite precipitation, while carnallite does not reach is precipitation point, our calculations predict that at water activity of 0.55, water volume will reach to 11 km^3^. Water activity-Volume relations are demonstrated in Fig. 2.

## Summary and conclusions

(1) The relation between water activity and relative humidity above the Urmia Sea is used to estimate the level, volume and lake during is evaporation.

(2) The computed Urmia Sea dimensions due to evaporation subject to present - day climate are as follows:

1-Surface water level will decrease from 1275 m (in 1990) to 1270 m above mean sea level;

2-Water volume will decrease from 25 km^3 ^(in 1990) to 11 km^3^.

## Appendix 1

The precipitation -dissolution equilibrium of an ionic mineral is described by the formula:(A1)

Where *v*_M_, *v*_X_, z_M_, z_X _and n are the amounts and charges of cations and anions in a mineral molecule and the amount of water molecules in mineral molecule, respectively. A thermodynamic equilibrium constant, *K*_*sp*_, for this reaction is defiend as:(A2)

Where *m*_*M*, *sat *_and *m*_*X*. *sat *_are the molal concentration of cation and anion, respectively, in the liquid phase saturated with respect to the solid phase; and *γ*_M _and *γ*_X _are the conventional single-ion activity coefficients of cation and anion at proper concentrations, respectively;  is the water activity of the liquid phase:(A3)

Where *p *and *p*_0 _are the saturated vapor pressure of the solution and pure solvent at the same temperature, respectively; *ϕ *is the osmotic coefficient of a particular solution and  is the summation of molalities of all solute species, including ionic species and neutral substances in the solution. The saturation degree of a mineral is defined by the following equation:

Where the numerator of the equation is the product of the ionic concentrations in a particular system.

The general approach for the calculation of ionic activity and osmotic coefficients for multiple component electrolyte solutions was developed by Pitzer's scientific school [22-24]. This approach was based on a set of theoretically and empirically derived equations that account for the interactions between the particular ions present in the solution and for indirect forces arising from the ion-solvent interactions. The up-to date and complete equations for conventional single ion activity coefficients and water activity are:(A5)(A6)(A7)

The meanings and calculations of all parameters in Eqs. (A5), (A6) and (A7) can be found in Krumgalz and Millero [32,33], Harvie et al. [34], Pitzer[24], Krumgalz [35,36], Eqs. (A5), (A6) and (A7) take into consideration various interactions of opposite - charged ions, like-charged ions, triple ionic interactions, and asymmetrical electrostatic mixing effects. Throughout these equations the subscripts *M *and *c *refer to cations, similarly the subscripts × and a refer to anions. ,  and  indicate the summation of the properties of all anions, all cations and all neutral species, respectively.

The double summation indices ∑_c_∑_<c'_, ∑_a_∑_<a'_, ∑_n_∑_c_, ∑_n_∑_a _and ∑_n_∑_<n'_, denote the sum over all possible paris of variations of all cations, anions or their pairs with neutral species, respectively.

A thermodynamic model for water evaporation and mineral precipitation from natural brines based on Pitzer approach was developed for modeling Urmia Sea evaporation. The input data include ionic concentrations (in molal units), thermodynamic products of mineral solubility and Pitzer's ion interaction parameters. The products of solubility and Pitzer's ion interaction parameters are downloaded from the proper database within the model. At initiation, water activity of the brine at 25°C and the degrees of saturation of all possible minerals.

Three cases can then occur which are discussed below.

(a) Initial brine is under saturated and with respect to all minerals, i.e., and their degrees of saturation (*Ω*_*i*_) in the system are less than 1.0. The water evaporation is then simulated by removing H_2_O by iteration from the brine until saturation with respect to any possible mineral and is reached, *Ω*_*i *_= 1.0000 ± 0.0001.

(b) Initial brine is oversaturated with respect to any mineral (*Ω*_*i *_> 1.0001). In this case, the model simulates the process of mineral precipitation from this type of brine up to its saturation. At each step, the model calculates single-ion activity coefficients, water activity for the particular concentrations and the degree of saturation of all possible minerals.

The degrees of saturation are compared at each step and the mineral with the highest *Ω *value precipitates. The process is repeated for all other minerals with *Ω*_*i *_> 1 as well.

(c) Initial brine is saturated with respect to least one single mineral (*Ω*_*i *_= 1.0000 ± 0.0001). This case also includes the final stages of cases (a) and (b).

The developed model then simulates the process of water evaporation from saturated brine.

The developed model allowed us to the amount of water removed from brine, the amount of precipitated minerals and ionic concentrations of residual brine at each step of evaporation modeling.

## Competing interests

The authors declare that they have no competing interests.

## Authors' contributions

Nosrat Heidari, director of project, carried out calculations and participative treatment. Mina Roudgar, sampling and analyzing Neda Ebrahimpour, sampling and analyzing. All authors read and approved the final manuscript.
